# Archimetrosis: the evolution of a disease and its extant presentation: Pathogenesis and pathophysiology of archimetrosis (uterine adenomyosis and endometriosis)

**DOI:** 10.1007/s00404-022-06597-y

**Published:** 2022-05-21

**Authors:** Gerhard Leyendecker, Ludwig Wildt, Matthias W. Laschke, Gerhard Mall

**Affiliations:** 1 Dieburger Straße 209, 64287 Darmstadt, Germany; 2 Sistranser Straße 188, 6072 Lans, Austria; 3 Institut für Klinisch-Experimentelle Chirurgie, Universität des Saarlandes, 66421 Homburg, Germany; 4 Wiesenbacher Str. 10, 69151 Neckargemünd, Germany

**Keywords:** Endometriosis, Adenomyosis, Archimetrosis, Pathogenesis, Primate evolution, Primary dysmenorrhea, Tissue injury and repair

## Abstract

**Purpose:**

This article presents a novel concept of the evolution and, thus, the pathogenesis of uterine adenomyosis as well as peritoneal and peripheral endometriosis. Presently, no unifying denomination of this nosological entity exists.

**Methods:**

An extensive search of the literature on primate evolution was performed. This included comparative functional morphology with special focus on the evolution of the birthing process that fundamentally differs between the haplorrhine primates and most of the other eutherian mammals. The data were correlated with the results of own research on the pathophysiology of human archimetrosis and with the extant presentation of the disease.

**Results:**

The term Archimetrosis is suggested as a denomination of the nosological entity. Archimetrosis occurs in human females and also in subhuman primates. There are common features in the reproductive process of haplorrhine primates such as spontaneous ovulation and corpus luteum formation, spontaneous decidualization and menstruation. These have fused Müllerian ducts resulting in a uterus simplex. Following a usually singleton pregnancy, the fetus is delivered in the skull position. Some of these features are shared by other mammals, but not in that simultaneous fashion. In haplorrhine primates, with the stratum vasculare, a new myometrial layer has evolved during the time of the Cretaceous–Terrestrial Revolution (KTR) that subserves expulsion of the conceptus and externalization of menstrual debris in non-conceptive cycles. Hypercontractility of this layer has evolved as an advantage with respect to the survival of the mother and the birth of a living child during delivery and may be experienced as primary dysmenorrhea during menstruation. It may result in tissue injury by the sheer power of the contractions and possibly by the associated uterine ischemia. Moreover, the lesions at extra-uterine sites appear to be maintained by biomechanical stress.

**Conclusions:**

Since the pathogenesis of archimetrosis is connected with the evolution of the stratum vasculare, tissue injury and repair (TIAR) turns out to be the most parsimonious explanation for the development of the disease based on clinical, experimental and evolutionary evidence. Furthermore, a careful analysis of the published clinical data suggests that, in the risk population with uterine hypercontractility, the disease develops with a yet to be defined latency phase after the onset of the biomechanical injury. This opens a new avenue of prevention of the disease in potentially affected women that we consider to be primarily highly fertile.

## Introduction

The essential aspects of a new view of the pathogenesis and pathophysiology of endometriosis have been presented in several publications by us since the mid-1990s of the last century [1–7]. It is the notion that in spontaneously occurring endometriosis and adenomyosis, ongoing muscular activity of the uterus [5, 8–11], especially during an ovulatory, non-conceptional cycle, leads to self-injury (auto-traumatization) and a healing process (tissue injury and repair; TIAR) [4, 5, 12] of uterine structures and parallel or subsequent dissemination of viable basal endometrial (archimetral) tissue fragments and stem cells (endometrial, ESC or archimetral, ASC), or archimetral progenitor cells, via the tubes into the abdominal cavity and via the vascular system into the periphery of the body [9, 13–15]. At these extra-uterine sites, biomechanical mechanisms of injury and repair [4, 5] are also operative and maintain the proliferative process.

The prevalence of primary dysmenorrhea as a clinical sign of uterine hypercontractility amounts to 50–60% of all young women [7, 17, 18]. However, menstrual bleeding has been very infrequent in the population of young women that drove reproduction for millions of years [7]. Thus, the potentially destructive effect of uterine hypercontractility, as observed today in many non-conceptual cycles, did rarely occur and, therefore, had no impact on reproductive biological evolution. In the human, the stratum vasculare constitutes the main muscular layer of the uterus and the contraction of this layer presumably causes primary dysmenorrhea [3, 5, 19–21].

Endometriosis is also described in subhuman haplorrhine primates exhibiting spontaneous decidualization followed by menstruation in non-conceptive cycles [22, 23]. The Haplorrhini separated from the Strepsirrhini with a common primate ancestor before the Cretaceous–Paleogene boundary (K–T boundary) [24]. Therefore, the assumption has to be made that auto-traumatization by hypercontractility is presumably also operative in all anthropoids.

**Fig. 1:**
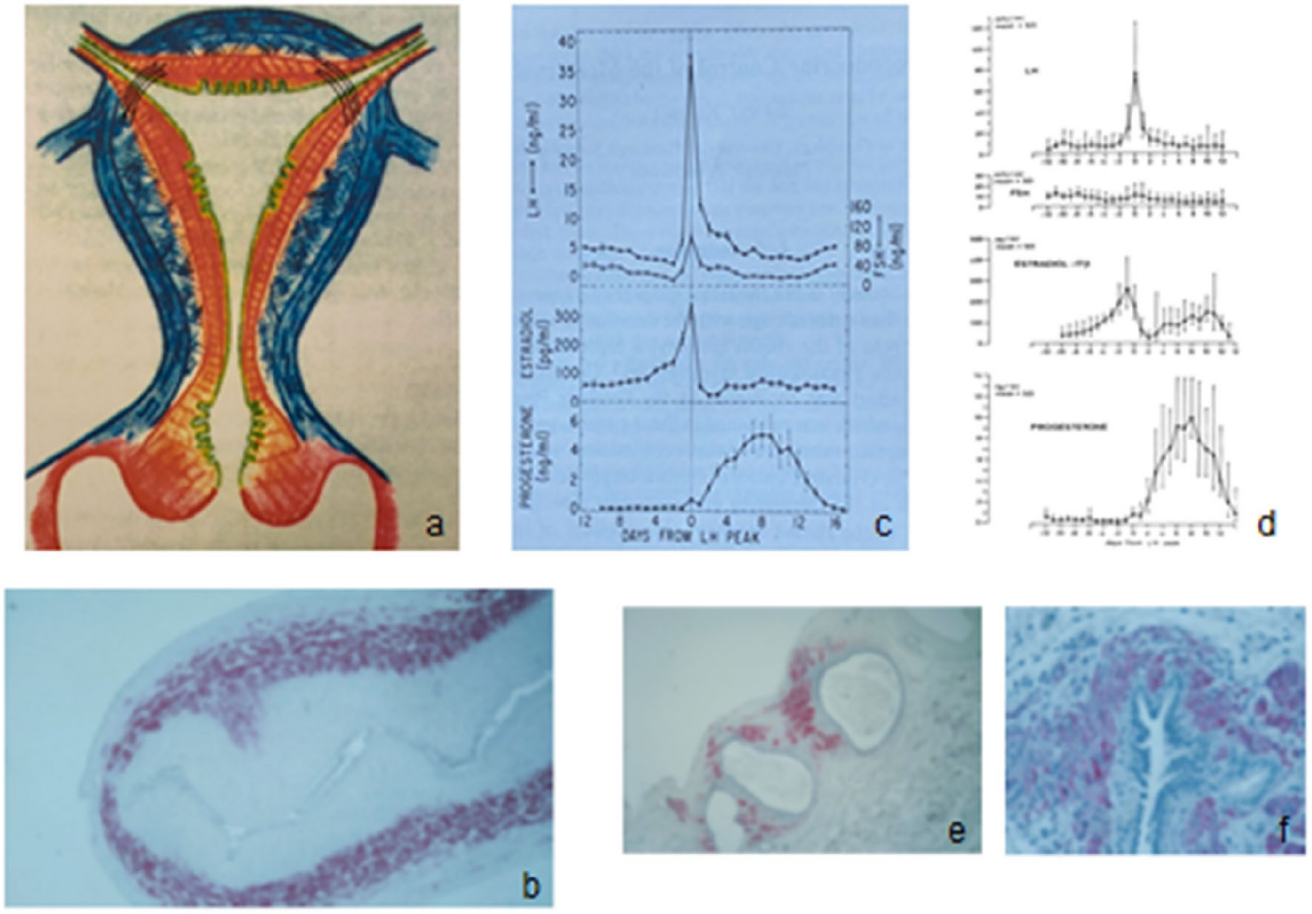
**a** Schematic depiction of the haplorrhine primate uterus (modified from [16]). Archimetra: *Paramesonephric (Müllerian) origin;* epithelial endometrium (green); stromal endometrium (orange); stratum subvasculare (orange) with predominantly circular muscle fibers. Function: peristalsis and antiperistalsis. The archimetra is the adult representation of the primordial uterus. Neometra: *non-Müllerian origin* (*blue*). Stratum vasculare (three-dimensional short muscular bundles with circular bundles around the intramural part of the tubes). Function: concentric contraction, hydrostatic pressure. Stratum supravasculare (with predominantly longitudinal fibers): function: fixation of the uterus to the pelvis. **b** The primordial uterus in the 27th week of pregnancy. Pituitary gonadotropins (LH, FSH) and ovarian steroids (estradiol and progesterone) in **c**. The rhesus monkey menstrual cycle (modified from [136]), **d** the human female menstrual cycle (modified from [227]). **e**, **f** Peritoneal archimetrotic lesions: Being composed of all tissue structures of the archimetra (epithelium, stroma and metaplastic myometrium), they can be considered as archimetral (“Müllerian”) organoid structures (modified from [15])

While it is well known that the myometrium of most placental mammals is composed of two myometrial layers [25, 26], the inner circular and the outer longitudinal layer, there is no direct information available on the myometrial structure in subhuman primates. It is very similar or even the same in all haplorrhine primates (Fig. 1). The exact knowledge, however, is of enormous importance for the understanding of the evolution of the haplorrhine primates in general [27] and the pathogenesis of archimetrosis in particular.

We suggest replacing the terms endometriosis and adenomyosis by the more comprehensive term ‘archimetrosis’, because we are dealing primarily with a disease of the archimetra [2, 6, 7, 13, 20] as well as proliferation of ASC and progenitor cells (genitoblasts) [15, 27–29] at the sites of primary lesions and parallel and subsequent trans-tubal [30] and vascular dissemination into the abdominal cavity and to the body periphery, respectively [15]. Archimetrotic foci within and outside the uterus are considered as archimetral (Müllerian) organoid structures that are composed of all the tissue elements of the primordial uterus [6, 7, 15, 31–33] (Fig. 1).

There is no doubt that non-conceptional cycles, uterine hypercontractility [1, 5, 15], spontaneous decidualization [23, 34], and menstruation constitute central aspects in the pathophysiology of the disease process. These phenomena have to be discussed in a broad evolutionary, primatological, historical, sociological and clinical context to be fully understood.

## Material and methods

### Concept

The pathogenesis of spontaneous archimetrosis (uterine adenomyosis and endometriosis) is unknown. This work is based on own previous results and therefore on the premise that archimetrosis is caused by tissue injury and repair (TIAR). It is aimed to corroborate this concept and elucidate the pathogenesis of the disease.

### Methods

An extensive search of the literature on primate evolution was performed. This included comparative functional morphology. Special emphasis was laid on the evolution of the birthing process that fundamentally differs between the haplorrhine primates and at least most of the other eutherian mammals. The data were correlated with the results of older and recent research on the pathophysiology of human archimetrosis and with the extant presentation of the disease.

## Historical overview and state of research

The honor of having rendered the first detailed description of the disease under discussion goes to Karl von Rokitansky [35–38]. At his time the term ‘sarcoma’ did not have the connotation of a malignant tumor [39–42].

About 30 years later, Friedrich von Recklinghausen [43, 44] and Wilhelm Alexander Freund [45] resumed the clinical and morphologic workup of these tumors, which they termed adenomyomas of the uterus.

Wilhelm Alexander Freund had the reputation as a brilliant gynecologic surgeon and was the first to publish a systematic and reproducible method of abdominal hysterectomy [46]. He realized that some of the benign uterine tumors he often was confronted with differed in many respects, both, clinically and morphologically, from uterine fibroids. He felt that he was dealing with a new, hitherto undefined gynecologic disease entity. He was able to make the correct diagnosis preoperatively and invited von Recklinghausen [44] into the operation theater during the operation of such a patient to demonstrate the *situs* and hand over the specimen for pathological workup [45].

As documented by their published cases, they were confronted with the full spectrum of the clinical picture of archimetrosis. It ranged from uterine tumors, foci on the pelvic floor, the uterine serosa and peritoneum, to ovarian tumors that, when ruptured, emptied "chocolate-colored" fluid [45]. Clinically, the patients presented with lower abdominal tumors of greater or lesser size, accompanied by pain, bleeding disorders and secondary sterility as the predominant symptoms. But primary sterility was also frequently observed. The triad of symptoms of endometriosis (archimetrosis), such as pain, bleeding disorders and sterility, was for the first time formulated by Freund [45].

It was Tomas S. Cullen [47] who was able to demonstrate a continuity of the glandular elements of the adenomyomas with the endometrial surface of the uterine cavity and that therefore these tumors were derived from the paramesonephric (Müllerian) ducts. He was familiar with the controversy between Recklinghausen's Wolffian duct hypothesis [43, 44] and the Müllerian duct hypothesis advocated mainly by Kossmann [37, 44, 48]. His first monograph entitled "Adeno-Myoma des Uterus" [49] was part of a ‘Festschrift’ for Johannes Orth of Göttingen, which he later extended into the English version [50]. In his review paper [51], he summarized the results of his many years of study of adenomyomas and their spread into the body.

The broad clinical and scientific interest in the adenomyoma of the uterus at the end of the nineteenth century [44, 47, 51] was the starting point for the still ongoing research on the pathogenesis and pathophysiology of ‘endometriosis’. Against this background, it is astounding that Sampson [37, 52] made the bold remark that uterine adenomyosis had no pathogenetic connection whatsoever with endometriosis [30]. This had an enormous (worldwide) impact on the perception of the disease [53, 54] and further research, which was recently mainly directed in identifying abnormalities in the “omics” (proteinomics, genomics, epigenomics) of endometrium of affected women [55–68] in comparison to normal endometrium. These are, in our opinion, secondary phenomena to the primary and evolutionarily highly conserved physiological process of tissue injury and repair (TIAR) [4, 6, 12, 69, 70] taking place at the level of the uterus.

Sampson’s concept of transtubal dissemination of cells and fragments of menstrual debris is widely accepted. His theory of simple retrograde menstruation causing endometriosis was only later questioned by the finding that transtubal flow of blood into the peritoneal cavity was apparently a physiological phenomenon [71, 72], which was immediately refuted by experimental data [73]. Philipp and Huber [74] came to the conclusion that the cells that cause endometriosis by transtubal transmission must come from deeper layers of the endometrium, rather than the functionalis shed during menstruation. A surprisingly modern aspect was presented by De Snoo [27], in that only cells that were involved in embryogenesis of the Müllerian ducts and in the regeneration of the endometrium after pregnancy could have the potential of causing endometriosis. He termed these cells ‘genitoblasts’.

In addition, Robert Meyer [75] criticized the term endometriosis, because it omitted the fact that all endometriotic lesions are composed of all Müllerian duct morphological elements, such as endometrial epithelium, stroma and metaplastic myometrial cells [15, 32, 33] (Fig. 1).

The original view that endometriosis and adenomyosis constitute a nosological entity, however, was not completely abandoned [36, 52, 74, 76–78] and was re-enforced by our own and other studies [2, 4, 5, 9–11, 13–15, 79–83].

Endometriosis (archimetrosis) in subhuman primates, anatomically and clinically, appears to be identical to the human disease and there is no evidence for suspecting a pathophysiological difference [84]. Furthermore, the disease goes far back into primate evolution.

## Results and discussion

Spontaneous archimetrosis has been described in subhuman primates [85, 86]. Meanwhile, archimetrosis has been found in all Great Apes, Old World and New World monkeys [87–94]. A high prevalence of archimetrosis is observed in primates kept in captivity and prevented from reproduction [95].

The separation of the rhesus monkeys from the main stem line leading to Homo took place about 25 million years ago, and that of the New World monkeys about 35–45 million years ago. The latter probably came through drafting on vegetation islands in the course of the continental drift and low sea levels from Africa to South America [96, 97].

This allows the conclusion that morphological, structural and functional predispositions for the development of archimetrosis were presumably already present in all Anthropoidea (haplorrhine monkeys). These primates menstruate following spontaneous decidualization in non-conceptive cycles. Thus, on the basis of present knowledge spontaneous archimetrosis constitutes an anthropoid primate disease. In this regard, data are lacking with respect to other known menstruating non-primate species, such as the spiny mouse (Acomys cahirinus), chiroptera and the elephant shrew (Elephantulus myurus) [98–101].

### The myometrium in Glires and Ferungulata

In the Ferungulata and the Glires, the myometrium is composed of two layers [102]. While the inner layer with circular (Müllerian) fibers subserves the ancestrally old peristaltic and anti-peristaltic functions of gamete and embryo transport [103], the outer longitudinal layer serves to support the forces for expulsion of the fetus or fetuses [13, 20]. Muscular connections between the two layers have been described in mice. They are thought to serve signal transduction and the synchronization of peristaltic contractile activity of both layers during delivery and, in the immediate postpartum period, for reduction of uterine blood minute volume that is increased in all placentals during and particularly at the end of pregnancy [104, 105].

Glires, such as the rabbit, which, like haplorrhine primates, have ancestral hemochorial implantation [106], can bleed profusely during delivery. Ferungulata have replaced ancestral hemochorial in favor of epitheliochorial (Ungulata) and endotheliochorial (Carnivora) implantation [102]. They do not bleed or only show some sanguinolent secretion after the expulsion of the afterbirth. In all placentalia, uterine contraction postpartum provides the conditions for the onset of efficient and permanent hemostasis by blood clotting [107].

### The shape and the myometrial composition of the uterus in menstruating non-human species

Menstruating higher mammals, such as the Old and New World monkeys, ovulate spontaneously and have a hemochorial implantation. Usually they have a uterus simplex. This also applies to the menstruating chiroptera with one exception [98, 99, 108]. The spiny mouse and the elephant shrew have a bicornuate uterus [23, 100, 101]. Menstruating mammals usually give birth to singletons [102, 109]. Exceptions to this are the spiny mouse and elephant shrew with two to four newborns.

Through the analyses of menstruating mammals, it is hoped to obtain suitable animal models for the study of human reproductive diseases, such as endometriosis and early pregnancy loss [34, 101, 110]. Although the gross forms of the uteri (uterus simplex versus bicornuate uterus) are generally well described, no information is available on the myometrial structure of the uterus in these animals. Surprisingly, this holds also true for the myometrial composition of the uterus in subhuman primates, although they are intensively discussed as animal models for the study of archimetrosis [111–115].

Thus, the exact knowledge of the myometrial structure in subhuman primates and how and why it evolved is lacking. With respect to the evolution of spontaneous archimetrosis, an analysis of the myometrial functional structure of subhuman primates and their evolution appears to be an important research desiderate.

### The composition of the myometrium in the human female

There is largely consensus on the three-layered nature of the human myometrium: the stratum subvasculare, vasculare and supravasculare [116, 117]. However, in the Anglo-American literature, Kreitzer’s terminology is usually not used [116–120]. The layers are named according to the predominant course of their fibers such as ‘criss-cross’ for the stratum vasculare. The functional significance of this specific fiber course of the stratum vasculare is apparently not widely appreciated.

Wetzstein [19] was the first to point out that, due to its composition of short muscular bundles, the stratum vasculare does not contract peristaltically, such as the other layers, but contracts in a concentric fashion in toto*.* Thus, during a concentric contraction the pressure increases within the entire uterine cavity.

**Fig. 2:**
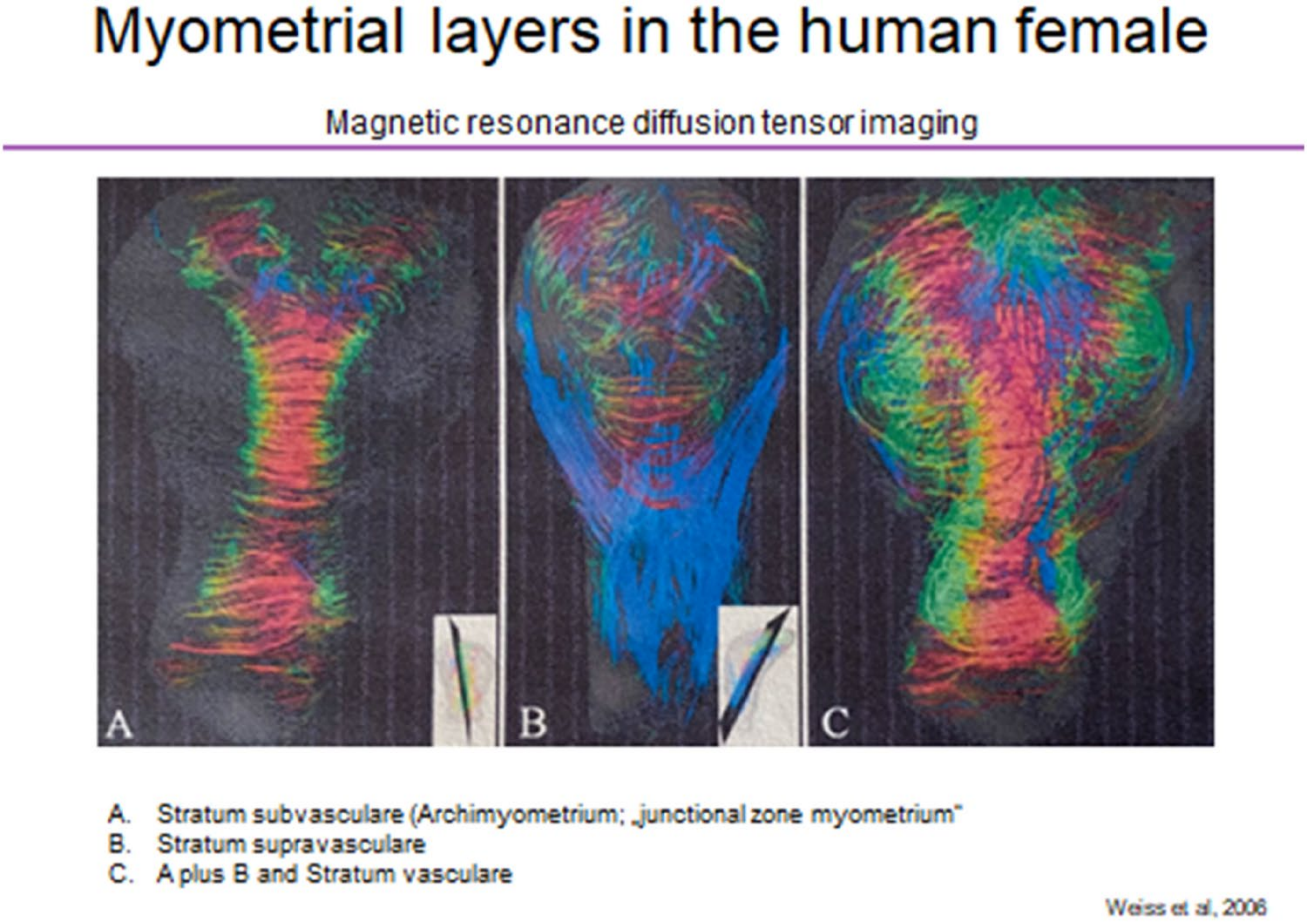
Magnetic resonance diffusion tensor imaging of the nonpregnant human uterus. **A** The stratum supravasculare with the fused Müllerian ducts displaying predominantly circular fibers. **B** The stratum supravasculare with longitudinal fibers (blue). **C** Aa composite of A plus B with the stratum vasculare. The stratum vasculare is composed of a three-dimensional net of short muscular bundles confined to the uterine corpus (with permission from [21])

In women, the main mass of the myometrium consists of the stratum vasculare, while the stratum supravasculare is largely regressed, as shown by magnetic resonance diffusion tensor imaging [21] (Fig. 2). This study also revealed an important structural detail of the stratum vasculare that may contribute to the delay between onset of the potential archimetral injury and the manifestation of the peritoneal disease [121, 122]: the short bundles of the stratum vasculare attain a more circular arrangement around the intramural part of the tubes. Thus, this part of the tubes is occluded, when the stratum vasculare contracts during parturition or menstruation (Fig. 1a).

### Peristaltic and aperistaltic birth

There is only indirect evidence that presumably all Haplorrhini (Anthropoidea) have, like the human, developed a stratum vasculare of the myometrium. This can be derived from studies that were performed by De Snoo in the Javan monkey (*M. fascicularis*) and the domestic pig (*Sus scrofa domesticus*) [27]. On the basis of film recordings during cesarean sections, he could demonstrate fundamental differences of the biomechanical birth processes between the primate and the pig.

In the Ferungulata he described the muscular biomechanics of the uterus during delivery as "peristaltic", while he described those in haplorrhine primates, quite similar to the concentric contractions during delivery in the human, as "aperistaltic". In the Ferungulata, the fetuses are literally pushed outward by peristaltic contraction waves of the myometrium, while in the haplorrhine primates the birth of the fetuses takes place by intermittent-rhythmic increases in intrauterine hydrostatic pressure.

Since Wetzstein [19] had recognized that the stratum vasculare is capable of strong concentric contractions due to its three-dimensional structure of short muscle bundles that simultaneously involve the entire muscle, we conclude that the aperistaltic birth in primates, as described by De Snoo, obviously constitutes the functional consequence of the specific structure of the stratum vasculare. Thus, the myometrium of subhuman primates appears to be composed, as that of the human female, of an inner circular layer (*stratum subvasculare; archimyometrium; junctional zone myometrium*), an outer longitudinal layer (*stratum supravasculare*) and a third layer in between, the *stratum vasculare*. Surprisingly, in his studies on the aperistaltic birth process in primates, De Snoo did not mention the stratum vasculare as the morphological basis of this phenomenon. This was probably due to Goerttler’s then famous and enforced concept of the structure and functioning of the human myometrium as a double spiral muscular tube [123] that prevailed until it was fully refuted by Wetzstein’s studies [19].

As mentioned above, muscular fibers and even muscular bridges in rodents serve to synchronize the peristaltic activity of the circular and longitudinal layers during parturition. It is reasonable to assume that these muscle fibers and muscle bridges between these two layers [105] constitute the *anlage* for the evolutionary development of the stratum vasculare in haplorrhine primates.

### Encephalization

The question arises as to why in Haplorrhini, with the stratum vasculare, a third, phylogenetically and ontogenetically young muscle layer [20, 116] developed at all and along with it the 'aperistaltic' birth [27], although in many and also large mammals the synchronized peristaltic muscular power of the circular and longitudinal layer is sufficient for successful completion of a birth. The answer lies in the need for stronger muscular strength in the birthing process in the Haplorrhini, of which the prenatal development is characterized by a preference for brain and therefore head growth over body growth [124]. This increased encephalization is already present in the haplorrhines after their separation from the strepsirrhines [125].

**Fig. 3:**
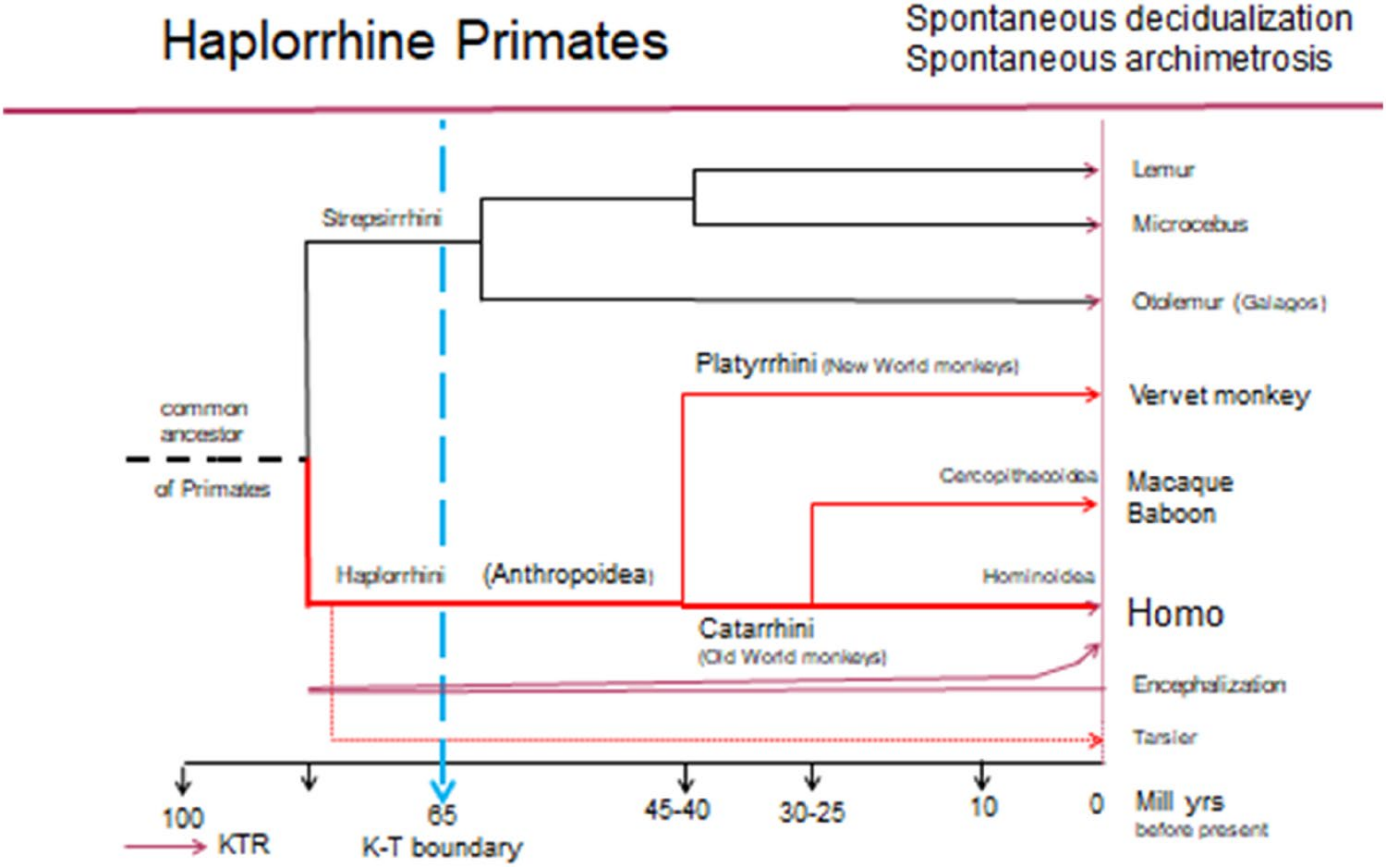
The evolutionary tree of the strepsirrhines and haplorrhine primates is shown. KTR: Cretaceous–Terrestrial Revolution. K–T boundary: time of mass extinction. During the KTR, the haplorrhines developed increased encephalization with predominant optical perception, a stratum vasculare, a uterus simplex, spontaneous ovulation, spontaneous decidualization, singleton pregnancies with delivery in skull presentation, and menstruation in non-conceptive cycles. In this suborder of primates, spontaneous archimetrosis was demonstrated

During the time of the Cretaceous–Terrestrial Revolution (KTR) [126], which ranges from about 120 million years ago to the Cretaceous–Tertiary boundary (K–T boundary) 65 million years ago, previously non-existent habitats had opened up in the course of the continental drift and with the appearance of a new, angiosperm flora, which, as a result of a mosaic of different mutations, led to a splitting of the early Eutheria in the sense of the favored "long fuse model" [127–131]. Thus, probably even before the K–T boundary, the pro-simians (Strepsirrhini) and the Haplorrhini (Anthropoidea) had developed from a common ancestor of the primates [102] (Fig. 3). As a result of their specific locomotion (grasping, climbing and jumping) as well as primarily optical detection of food and, thus, due to the preference for optical over olfactory sensory perception of the environment, the early anthropoids were able to assert themselves as diurnally active animals in the canopies of tropical forests as an arboreal habitat. The preference for optical perception to the detriment of olfactory perception resulted in an increase in the neocortex and, thus, the beginning of an increased brain growth that accelerated in the apes and became explosive only in the last 2–3 million years [97, 124, 132].

Due to a mosaic of parallel, evolutionary processes [24], it was ensured that singleton pregnancy and birth in skull position became the norm. Already, De Snoo points out that all anthropoids take a sitting or squatting position at rest and in sleep [27, 97]. Thus, the head of the fetus can enter into a firm relationship with the pelvis prior to birth [27].

The Tarsier are haplorrhine and considered a sister taxon to the anthropoids [133]. Their eyes are bigger than their whole brain. This and some differences in the neural organization of their optical sensory system may be attributed to the fact that they probably secondarily changed from a diurnal to nocturnal activity [134, 135]. They have a uterus simplex and singleton pregnancies. In our context, it is of interest that these animals are forced into a permanent vertical posture, which has to be made possible for them when they are kept in captivity.

The entire female reproductive system of primates, including specific structure and function of the myometrium [19, 27], had principally already reached the stage of development as in humans, when the Cercopithecoidea were separating off the stem line leading to Homo, if not before (Figs. 1, 3). Spontaneous alternating ovulations on the basis of a “permissive” hypothalamic GnRH secretion [136–143], a uterus simplex with a fundo-cornual raphe [14, 116], menstrual bleeding after spontaneous decidualization [23] in all Haplorrhini possibly including the tarsiers and the occurrence of archimetrosis in all Anthropoidea allow the conclusion that the specific development of the reproductive system of haplorrhine primates represents a predisposition to the development of archimetrosis.

**Fig. 4:**
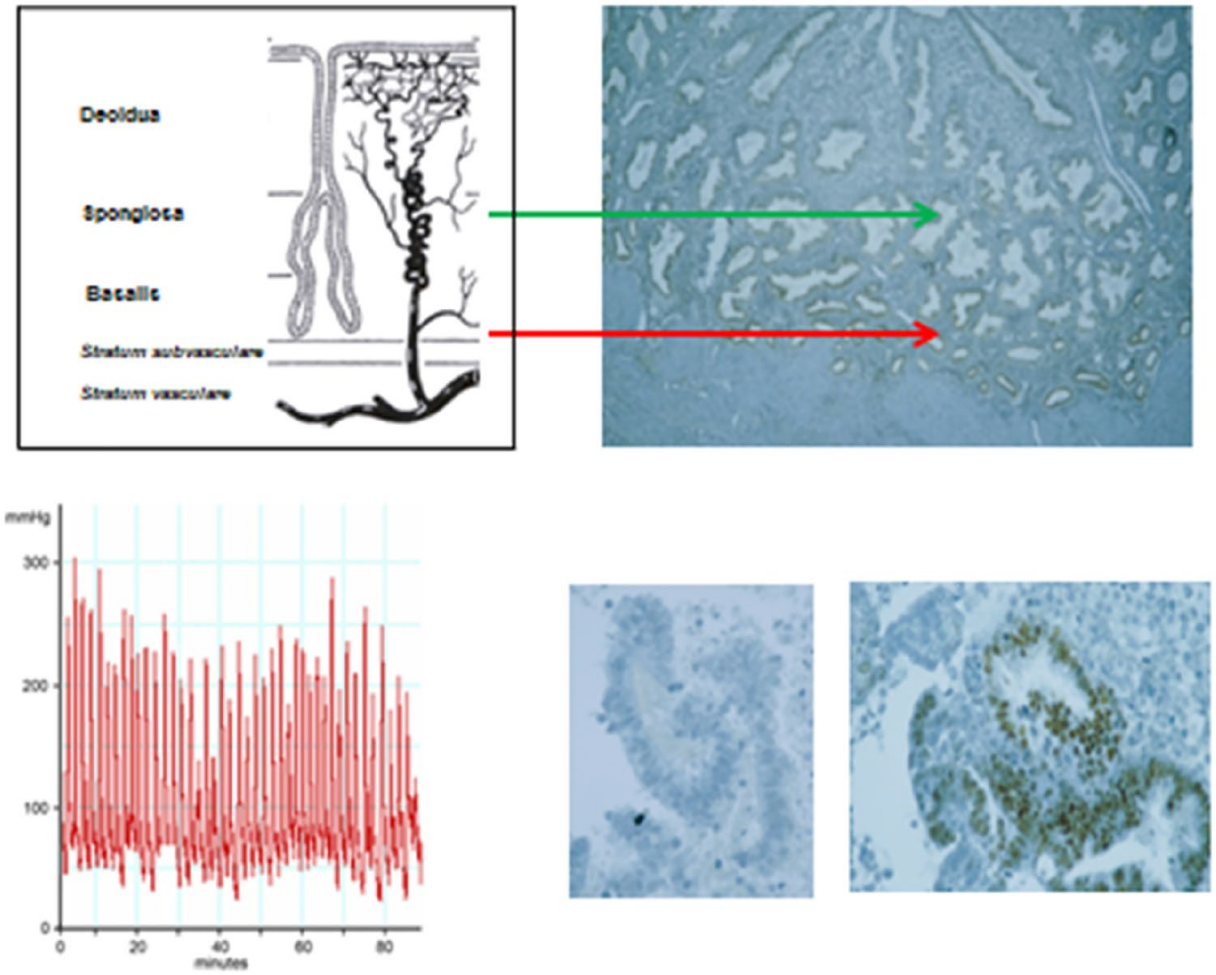
This is a schematic demonstration of the level of detachment of archimetral tissue in healthy women and in women with uterine hypercontractility and archimetrosis. *Healthy women:* In women with no primary dysmenorrhea or only little discomfort during menstruation, the functionalis is detached on the level of the spongy layer of the endometrium. The decline of progesterone in a non-conceptive cycle results in a progressive shrinkage of the functionalis and a concomittant compression of the spiral arteries. Due to the blood supply of the functionalis by arterioles branching off from the spiral arteries, ischemia occurs only in the functionalis and in the upper spongiosa layers, while the blood supply of the basalis with arterioles branching off from the radial arteries is unaffected. Menstrual blood contains only fragments of menstrual debris with no vital cells. The cells are not vital, because of a longer process of increasing ischemia. The menstrual blood coming from the venous lacunae in the upper functionalis is dark, watery and not clotting (level of detachment: green arrow). As a physiological process, menstruation in healthy women does not constitute tissue injury. *Women with hypercontractility, severe and extreme primary dysmenorrhea:* While the shrinkage of the functionalis and the compression of the spiral arteries have already commenced during the late luteal phase with the onset of severe menstrual contractions of the stratum vasculare also the radial arteries and the branching arterioles that supply the basal layer of the endometrium are compressed. This results in acute intermittent ischemia. Fragments of vital basal endometrium with archimetral stem or progenitor cells are detached. Menstrual blood is now mixed with fresh blood coming from the basal arterioles; it becomes more reddish and may coagulate. This is acute injury to the archimetra that induces the process of tissue injury and repair (TIAR) (modified from [15] and [228])

In Old World monkeys, birth is often a long-lasting, difficult biomechanical process. With the relatively wide pelvic entrance in the large apes, the birth process seems a bit more effortless [97]. Irrespective of the various theories behind the initiation of the birth process [144–146], after the explosive encephalization in Homo the need for the birth of a largely immature newborn arose. Thus, for the birth and control of the postnatal period, a large concentric contraction force that is provided by the stratum vasculare represented an evolutionary advantage, and it has to be kept in mind that in the wild and also in archaic human societies non-conceptive cycles and menstruation were and still are rather infrequent incidences [7]. However, in the non-conceptual cycle, uterine hypercontractility leads to biomechanical stress and traumatization of deeper layers of the archimetra obviously in all anthropoids (Fig. 4). In some colonies of macaque (*Macaca Fuscata*), the prevalence of acquiring archimetrosis in captivity with prevention of conception is as high [95] as the prevalence of severe and extreme primary dysmenorrhea in young women [16, 147].

Against this evolutionary background, it will be examined to which extent anamnestic, clinical, histological and immunohistochemical data as well as data obtained by imaging techniques of the extant disease in women are compatible with the concept of auto-traumatization of the uterus exerted by its own biomechanical functions.

## Primary dysmenorrhea

Uterine contractions at the end of an ovulatory cycle and labor pains are homologous to each other in their initiation (decline of progesterone blood levels) and functionality (emptying of the uterus). Primary dysmenorrhea reflects increased contractility of the stratum vasculare; it occurs only after ovulatory cycles [148, 149].

Amounting up to 50–60%, the prevalence of primary dysmenorrhea in young women is high [16]. Instead of a pain score, we used in our studies easily remembered anamnestic data to assess the severity of primary dysmenorrhea. We do not consider the more or less mild abdominal pulling that many women experience with the onset of menstruation as dysmenorrhea. Our classification is based on the analgesic requirement [4, 7]:

*Mild primary dysmenorrhea*: painful contractions that are tolerated without analgesics.

*Moderate primary dysmenorrhea*: analgesics are occasionally required.

*Severe primary dysmenorrhea*: persistent perimenstrual need for analgesics.

*Extreme dysmenorrhea*: absence from school and work (absenteeism).

Particularly women with persistent severe and extreme dysmenorrhea are at high risk of developing archimetrosis. Severe forms of uterine archimetrosis, cystic–cornual angle adenomyosis (“zystische Tubenwinkeladenomyose”) [44], also termed salpingitis isthmica nodosa [75], were found only in women with extreme dysmenorrhea [5]. However, even (possibly) asymptomatic women develop archimetrosis over time. Ten years after the last birth, on the occasion of tubal sterilization, approximately 30% of women had peritoneal archimetrosis. The percentage decreased with a decreasing time interval from the last birth [121, 122]. Thus, there appears to be a strength–duration relationship between contraction strength and number of menstrual cycles, which in combination lead to archimetrosis. The circular structure of the muscular fibers of the stratum vasculare just around the intramural part of the tubes [21] may impede and delay the transtubal dissemination of archimetral stem cells into the peritoneal cavity. In any event, almost all women develop perimenopausal adenomyosis (archimetrosis), and 70% of women, who underwent post-mortem examination, had adenomyosis according to classical pathologic–anatomic criteria. Since confirmed peritoneal endometriosis (peritoneal archimetrosis) is often no longer detectable in women in late post-menopause and old age, uterine adenomyosis (uterine archimetrosis) is in the foreground owing to persistent structural changes of the uterus [36, 150].

## Uterine contractility

Assuming a mean peristaltic activity of two contraction waves per minute throughout the proliferative phase in stable ovulatory cycles, 5–6 million contraction waves occur during the first 10n years of reproductive maturity, exerting their highest power in the fundal region of the uterine cavity [5]. Similarly, in the first 10n years after the onset of stable cycles, assuming 24–36 neometral contractions per hour, which last for about 36 h with decreasing strength, approximately 110–140 thousand compressions of the archimetra occur during menstruation by the concentric contractions of the stratum vasculare. Due to the distribution of uterine muscle mass, these also develop their strongest force in the fundal region of the uterus [5].

### Hyper- and dysperistalsis

In women with endometriosis/adenomyosis (archimetrosis), the mean number of contraction waves is doubled and the intrauterine pressure is increased [1, 8, 83]. This leads to permanent traumatization at the fundo-cornual raphe of the archimyometrium [2, 14, 116]. In our opinion, this is a predilection site for the development of uterine archimetrosis, since the peristaltic contractions associated with unilateral directed sperm transport can lead to a chronic, cyclically changing, asymmetric mechanical stress and, thus, injury to the stromal fibroblasts at this dividing line of the formerly separated two Müllerian muscle tubes (fundo-cornual raphe) [1–8, 14]. Early focal uterine archimetrosis is often observed with magnetic resonance imaging (MRI) in the midline fundal part of the archimyometrium [9, 10].

Hyperperistalsis probably develops already before menarche when estradiol levels are rising and falling during "occult anovulatory cycles", in which follicles can grow to preovulatory size [151–155]. It may lead to injury and desquamation of cells and fragments of the basalis and, thus, stem cells because this layer is not yet sheathed by substantial proliferation of the endometrial mucosa.

### Compression of the archimetra by the concentric contractions of the stratum vasculare

In our opinion, the essential auto-traumatization occurs due to the increased contractions of the stratum vasculare at the end of an ovulatory cycle, which initially manifest as primary dysmenorrhea [5]. Compression not only of the spiral arteries due to shrinkage of the functionalis, but also of the radial arteries occurs, resulting in intermittent acute ischemia of the basal and subbasal endometrium and its stroma and, in extreme cases, of the entire uterus [147]. Desquamation of deeper layers of the basalis ensues [5, 15]. Now, menstrual bleeding is accompanied by acute injury due to pressure and ischemia (Fig. 4). The blood that is dark, more watery, and non-coagulable during normal menstrual bleeding [156] becomes more reddish and may coagulate in parts since it is mixed with fresh blood from the deeper capillaries that supply the deeper part of the archimetra including the archimyometrium [157–159]. The intrauterine pressure may by far exceed the blood pressure in the arterioles (30 mmHg) during contractions and also between them, so that prolonged uterine ischemia occurs as the pathophysiological basis of extreme primary dysmenorrhea with severe vegetative symptoms and absenteeism [5, 147] (Fig. 4).

From a heuristic and terminological point of view, we do not concur with the notion that ‘normal menstrual bleeding’, such as described by Ober [156] and the subsequent regeneration of the functionalis, should be considered as a process of “Tissue injury and repair” [160]. As a physiological process, endometrial detachment during normal menstruation only involves the non-vital functional layer of the endometrium (Fig. 4).

There is indirect evidence that the hypercontractility of both, the stratum vasculare and the stratum subvasculare, in women with archimetrosis is associated with an increased oxytocin receptor expression [161–163].

## Uterine archimetrosis (adenomyosis)

According to the prevalent definition, uterine archimetrosis (adenomyosis) represents tumors consisting of endometrial glands proliferating into the depth of the uterus and hyperplastic *myometrium* [164, 165]. This definition, however, is inadequate, because it does not refer to the genuine character of hyperplastic archimetral myometrium and, thus, the nature of adenomyosis as an archimetral (Müllerian) organoid tumor with metaplastic (archimetral) muscle tissue [166]. Based on preliminary findings by Sitzenfrey [167], according to which smaller sub-endometrial lesions were much more frequently encountered in the surgical specimen than large isolated adenomyomas, Frankl [168, 169] coined the term adenomyosis, thus emphasizing the frequently disseminated character of the adenomyotic lesions. Bird and coworkers [164] took up this phenomenon again with the term subbasal adenomyosis.

Furthermore, adenomyosis could only be diagnosed definitively if the glandular proliferations would extend at least one simple field of view deep into the uterus or if the so-called junctional zone (JZ) was at least 12 mm thick on MRI [170, 171]. These criteria may also be based on the view [172] that "endometrium, wherever located, has an inherent proclivity to proliferate into subjacent tissues." Presumably, this view ultimately stems from Recklinghausen's rather casual comment that the chronic reproductive biologic stress on the endometrium, meaning pregnancy and childbirth, could be a stimulus to proliferation [44]. From this, and probably from some of the cases described by Recklinghausen and Cullen, stems the erroneous view that uterine archimetrosis is a disease of the multiparous older woman and has no pathogenetic similarities with peritoneal archimetrosis in the younger woman [173].

**Fig. 5:**
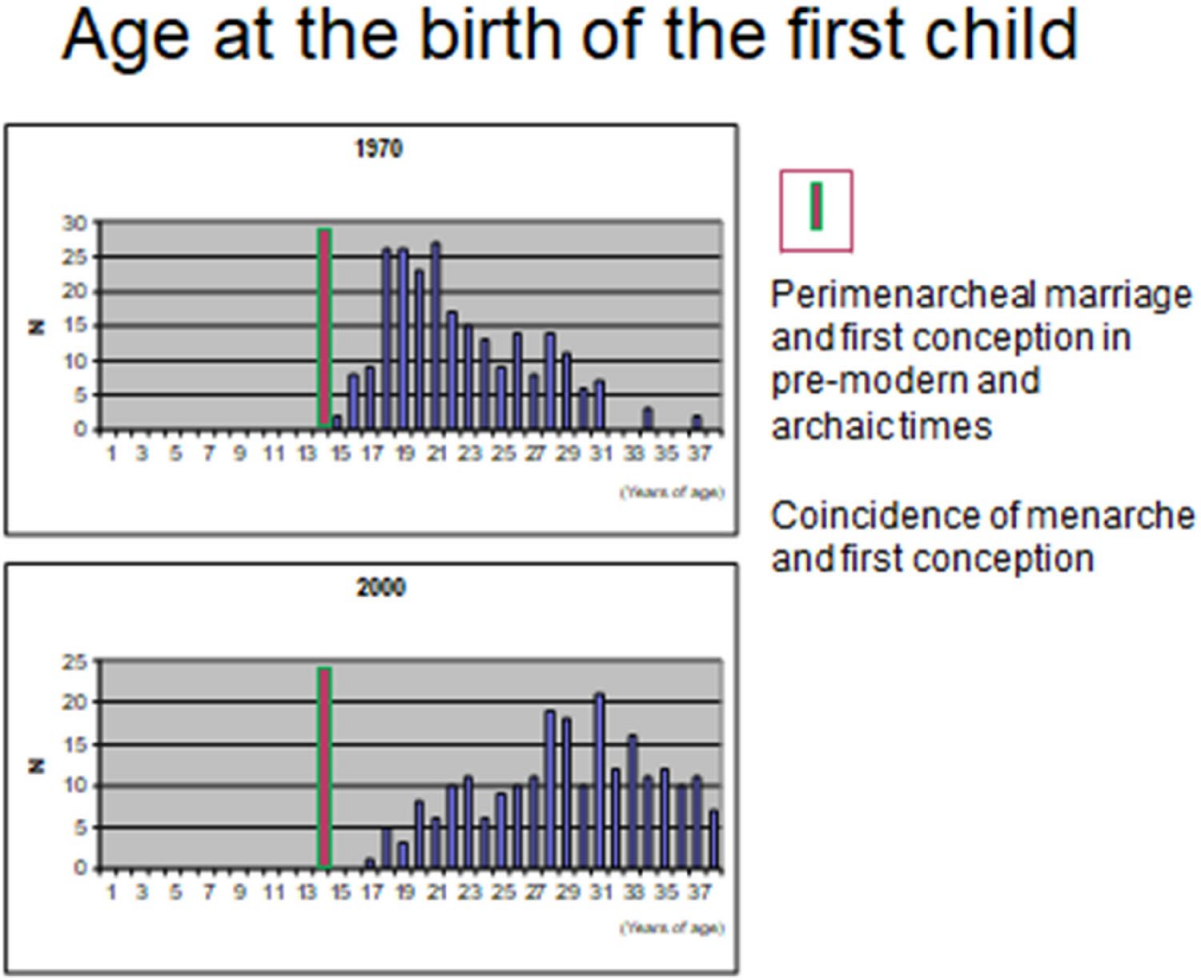
The age at the birth of the first child in the years 1970 and 2000, respectively, is shown. Data were obtained from the birth book of the Department of Obstetrics and Gynecology of the Klinikum Darmstadt, 63,283 Darmstadt, Germany, Teaching Hospital to the Universities of Frankfurt and Mannheim/Heidelberg. The column (red with green frame) at an age around 14 years is based upon historical data, historical holidays and rites that are connected with sexual maturation and fertility and go in part back into Neolithic times

There is no doubt that the disease on all levels, such as the uterus, the peritoneal cavity and the periphery of the body, takes time to develop probably dependent upon the severity of and the individual susceptibility to the biomechanical strain. Today, the patients often present with primary sterility, menstrual pain and severe discomfort [15, 62]. Two to four generations ago the patients presented at rather the same age often as parous women and with secondary sterility [45, 49] or the diagnosis was made at tubal sterilization of parous women [121, 122]. This pattern of presentation of symptoms allows the extrapolation of the reproductive potential of women with primary dysmenorrhea back into the early postmenarcheal period of life: they are very fertile. In fact, onset of regular ovulatory menstrual cycles indicates reproductive health (Fig. 5).

Undoubtedly, adenomyosis, like any neoplasm, begins as a very small lesion microscopically [50, 51]. In the now very extensive MRI literature [171, 174–178], focal widths of JZ as small as 6 mm are discussed as indicative of adenomyosis. We used in our studies a width of the JZ of 10 mm and additional visual criteria such as cyst formation to establish the diagnosis of adenomyosis on MRI. The same is true for high-resolution vaginal ultrasonography (VSG) [5, 9, 10, 79].

Hricak [174] could not clearly assign the low-intensity band she first described by MRI in the uterus to any anatomic structure. Tentatively, she used the term "endometrial–myometrial junction." Undoubtedly, the hypointense band-like structure with a mean width of about 5–6 mm in healthy woman [9] represents the stratum subvasculare of the myometrium that is morphologically characterized by little connective tissue and densely packed cells [179].

The 'expansion' of the JZ should be primarily regarded as a radiological phenomenon. The "broadening" of the JZ [165, 180] actually consists in the destruction of the archimyometrium in the early process [177] and its replacement by newly formed archimetral (Müllerian) muscular tissue. In early cases of development of adenomyosis (uterine archimetrosis) with proliferation of the endometrial glands and basal stroma into the deep layers, at first, a hyperintense disruption of the hypointense structure occurs on MRI. Subsequent stroma-muscular metaplasia then results in a widening of the junctional zone on MRI. This process of the development of uterine archimetrosis may be focally or diffuse [5, 9, 10].

### Tissue injury and repair

Hypercontractile leasioning of the archimetra with the desquamation of basal fragments initiates a physiological healing process that, as in other wounds in the body [181–186], involves the local upregulation of P450 aromatase and formation and paracrine action of estradiol as a process of tissue injury and repair (TIAR) [4] and, furthermore, the formation of the “morphogenetic complex” ERß/CXCL12/CXCR4 [6, 160, 187, 188]. Data from animal experiments support this view. In the urodele, it was shown that experimental heart lesions healed faster when estradiol was injected locally. This effect was mediated by the increased expression of CXCL12 [189].

The CXCR4 receptor on stem cells binds to the chemokine CXCL12, which is upregulated in the endometrium by high estradiol concentrations. There is an accumulation of mesenchymal stem cells in the wound area, which are converted into archimetral progenitor cells in the immediate apical vicinity of the epithelium, as the ligand, CXCL12, is formed in the endometrial epithelium [187, 190]. The attraction of stem cells takes place via the archimetral vascular system [159]. The dividing ESC (ASC) and progenitor cells are HOXA-10 regulated [191] and form endometrial epithelium at the apical parts of the glands through stroma–epithelial transformation and, thus, provide continuity between the original glandular tube and the glands of the newly formed organoid structure. At the opposite pole, through fibromuscular metaplasia, muscular tissue develops. Cullen [49] called these (Müllerian) archimetral structures “uteri en miniature”.

The paracrine action of *locally produced* estradiol within the TIAR process, together with ovarian estradiol, may further increase uterine contractility and, thus, the process of injury. It is not clear which factors are responsible in the individual case if and when an adenomyoma becomes arrested in its growth or if it develops in a monstrous uterine tumor of Müllerian tissue. Most likely, the severity and extent of the trauma is important in this regard as observed in extreme primary dysmenorrhea with the development of cystic cornual angle archimetrosis [5]. In iatrogenic uterine archimetrosis, we observed larger uterine lesions following post-partum curettage and curettages following miscarriages as well as after surgical abortion [75, 78].

The composition of glandular and muscular parts can vary considerably. Secretory and blood drainage from proliferated glandular tubes may be obstructed, resulting in blood-filled cysts that can be detected by hysteroscopy and transvaginal sonography [5, 192–195].

We do not concur with the notion to consider lesions of deeply infiltrating endometriosis (archimetrosis, DIA) that grow into the lower parts of the uterine corpus as a variant of uterine adenomyosis. By definition, they do not fulfill the developmental criteria of uterine adenomyosis (uterine archimetrosis) [196].

## Peritoneal and peripheral archimetrosis (endometriosis)

As already suggested by Philipp and Huber [74] and by De Snoo [27], the cells that cause peritoneal archimetrosis must come from a deeper layer of the endometrium and must have the potential of stem cells (genitoblasts). This has been experimentally substantiated by demonstrating that in patients suffering from peritoneal archimetrosis, basal endometrial fragments were detached during menstruation in a significantly higher proportion than in controls without the disease. From these data, it was concluded that in women with peritoneal archimetrosis basal endometrial fragments with stem cell character were shed via the tubes into the peritoneal cavity, where they implant and proliferate to archimetrotic lesions [15]. In such studies, it is often difficult but of paramount importance to recruit adequate young healthy controls with regular cycles, proven fertility, absence of primary dysmenorrhea and that of uterine archimetrosis as demonstrated by a normal stratum subvasculare (archimyometrium, “junctional zone myometrium”) by MRI or transvaginal sonography [197]. Infertility is often associated with focal adenomyosis [198].

Transplantation of the endometrium of mice into their peritoneal space initially leads to a seemingly complete destruction of the transplanted tissue with loss of the glandular structures within a few days. Only some days thereafter, apparently from surviving progenitor cells and along with the formation of vessels, a new archimetral structure with typical formation of glands develops at the site of the implantation [199].

In animal experiments, human transplanted archimetral stem cells (ASC) or progenitor cells regulated like the native tissue by HOXA-10 [191] form endometrial glandular epithelium by stromal–epithelial transformation and muscular tissue by fibromuscular metaplasia [28]. It is likely that after vascular or transtubar dissemination of the cells from the uterus as well as after their implantation in the periphery of the body or in the peritoneal cavity, the same processes of differentiation take place [29]. Accordingly, with the archimetrotic lesions Müllerian organoid structures develop that in principle have the same tissue composition as the primordial uterus or the later archimetra [15] (Fig. 1e, f).

Bird and coworkers [164] described in their study on patients undergoing hysterectomy that the most frequent menstrual disorders were not encountered in patients with extensive adenomyosis, but rather in women with subbasal lesions. Premenstrual and postmenstrual spotting is considered as a typical symptom of endometriosis [45]. Therefore, we consider that the seeding of vital endometrial fragments containing stem or progenitor cells may already or even preferentially occur in the early phase of the development of uterine archimetrotic lesions. It also has to be taken into consideration that due to increased intrauterine pressure and cervico-fundal peristaltic activity, which is already present at the end or immediately after the menstruation in women with peritoneal archimetrosis [1, 81, 82], proliferating progenitor cells of the early functionalis [200] are transported via the tubes into the peritoneal cavity.

The stratum vasculare as well as the stratum subvasculare (archimyometrium) are in the case of hypercontractility both involved in the transtubal dissemination of archimetral stem or progenitor cells.

The increased neometral contractions repeatedly lead to deep injuries of the endometrium with detachment of fragments of vital basalis, which contain archimetral bone marrow-derived stem cells due to the chronic process of wound healing (TIAR) and also resident progenitor cells and may be disseminated into the periphery of the body or by retrograde blood flow into the peritoneal cavity.

In addition, the increased peristalsis of the archimyometrium (hyperperistalsis) results in a permanent “Durchwalkung” (kneading) of the injured endometrium in cervico-fundal direction. This effect is enhanced when hyperperistalsis changes at mid-cycle and high estradiol levels into dysperistalsis with convulsive contractions of the entire uterus [1]. This constant kneading of the injured uterus through hyper- and dysperistalsis may contribute together with the hypercontractility of the stratum vasculare to the lymphatic dissemination of stem and progenitor cells into the periphery of the body. Accordingly, endometrial tissue has been detected in pelvic lymph nodes and the groin in patients with endometriosis [201, 202].

## Persistence of archimetrosis by biomechanical strain

### Predilection sites of extra-uterine archimetrosis

After Cullen's detailed description of the localization of extra-uterine archimetral foci [51], other sites of predilection, such as the retrocoecale area and the peritoneum of the diaphragm, have been further recognized. Dramatic catamenial conditions, such as recurrent pneumothorax or seizures, have shown that the pleura and brain, among others, may be involved [203–206].

The topography of the uterus, tubes, ovaries, and the other intraperitoneal and extra-peritoneal abdominal organs such as the intestine and urinary bladder is certainly of importance, in that increased menstrual detritus may collect in the dependent areas of the abdomen and in the various niches formed by different organs. However, the crucial criterion in both intraperitoneal and peripheral archimetrosis is not the implantation of endometrial epithelium and stroma as such, but the onset and persistence of the proliferative and inflammatory processes by stem and progenitor cells as a result of local trauma.

Thus, the question arises whether predilection sites of extra-uterine archimetrosis in the body are characterized by persistent biomechanical strain. This view appears to be supported by the localization of the lesions, as described by Cullen [51], and continues to be supported by newly described sites such as the pleura and the diaphragm [203, 204]. Deep infiltrating archimetrosis should therefore serve to enforce our view that the persistence and possibly progression of extra-uterine archimetrosis at whatever site in the body is supported by constant biomechanical strain.

### Deeply infiltrating archimetrosis (DIA)

Deeply infiltrating archimetrosis (DIA) is the most painful and debilitating variant of the disease and has been the subject of extensive discussion for decades regarding its development [207–209]. According to a proposed definition, deeply infiltrating archimetrosis (endometriosis) is present when the depth of infiltration of the foci exceeds 5 mm [65, 210]. Such foci preferentially start in the pouch of Douglas. From there, they may extend into the sacro-uterine ligaments, caudally into the cervix and the vagina, may penetrate into the rectum and obstruct the ureters. DIA may also penetrate into the lower parts of the uterine corpus and falsely considered as a special variant of uterine archimetrosis [196]. DIA is the subject of a special classification system, the Enzian classification [211–213].

Normally, the uterus and cervix are flexibly connected by the cervico-uterine junction. Movements of the cervix are therefore not readily transferred to the uterus, as any gynecological examination of a healthy woman will show. As soon as an archimetrotic focus infiltrates into the cervico-uterine junction from the pouch of Douglas, this structure becomes coarse and hard. The previously flexible cervico-uterine axis becomes rigid. It is not uncommon for the previously anteverted and anteflexed uterus to transition to an erected and stretched position. Both cervix and uterus become lever arms with the fulcrum of the lever at the suspension of the upper cervix and the now rigid cervico-uterine junction at the pelvic ligaments. That posits the fulcrum just inside the proliferative and inflammatory process. The movement of the cervix as the caudal lever arm during examination is painful and explains the patient's often extreme dyspareunia. The corpus uteri that rested before on the pelvic floor now constitutes the cranial arm of the lever and is inevitably moved during virtually any physiologic body activity, such as walking and even breathing. The result is a chronic TIAR process with proliferation and infiltration into the surrounding area. The first case of this type is described by Freund and v. Recklinghausen [44, 45].

On the basis of these considerations the following general view of the pathophysiology of peritoneal archimetrosis is proposed.

Following a TIAR process on the level of the basal endometrium, archimetral stem cells (ASC) or progenitor cells are transported to the extra-uterine foci and form archimetral organoid structures following the HOXA-10 program ("micro-primordial uteri") [15]. Like uterine archimetrosis, the peritoneal variety follows vascular structures, since only through them the attraction of stem cells occurs. Formation of capillaries [199] as well as sprouting of nerve fibers in and around the focus is massively promoted by estradiol-dependent growth factors, such as vascular endothelial growth factor (VEGF) and nerve growth factor (NGF) [32]. At sites without mechanical stress and therefore without the paracrine morphogenetic effect of estradiol, implanted endometrial tissue spontaneously regresses forming white fibrotic scars.

In the persistent archimetrotic foci, peripheral estradiol levels and paracrine estradiol concentrations appear to act additively. Reduction of either component (suppression of ovarian function or, for example, resolution of an adhesion between the intestine and uterus to terminate the local biomechanical irritation and TIAR process) may terminate the local proliferative and inflammatory process. Occasionally, with the administration of aromatase inhibitors [214], additional direct drug intervention in the TIAR process may be necessary to deprive the basal morphogenetic complex consisting of ER-beta, CXCL12, and CXCR4 of estrogenic supply.

### Cross talk between Müllerian organoid structures

Subfertility and infertility in the presence of minimal and mild peritoneal archimetrosis with patent tubes and unaffected tubo-ovarian complex has been considered as idiopathic, because operative or hormonal treatment did not result in a significant improvement of fertility [215, 216]. At the time of these studies, however, uterine archimetrosis had not at all been taken into consideration. Thus, in any event, removal of peritoneal lesions was widely not considered a successful option for improving fertility in patients suffering from peritoneal archimetrosis. Rickes and coworkers [217] for the first time demonstrated that in patients with severe peritoneal archimetrosis the pregnancy rate was significantly improved, if prior to in vitro fertilization (IVF) a systemic treatment with a long-acting gonadotropin-releasing hormone (GnRH) analog was performed. This positive effect on the pregnancy rate could not be demonstrated in patients suffering from minimal to mild peritoneal archimetrosis. Again, at that time uterine archimetrosis was not taken into consideration.

Surprisingly, radical surgery of peritoneal archimetrosis resulted in an improved pregnancy rate in spontaneous and IVF cycles in comparison to untreated patients. Furthermore, it could be demonstrated that “inflammatory” markers that could be detected in the peritoneal archimetrotic lesions as well as in the endometrium of the affected women had disappeared in the endometrium after the eradication of the peritoneal lesion. Unfortunately, whether at all and to what extent uterine archimetrosis was present in these patients was not documented by vaginal ultrasound or MRI in this study. On the basis of our own results, we assume that these patients had both, uterine and peritoneal archimetrosis [61, 218–220]

We would like to denominate this phenomenon as “Müllerian” crosstalk. A crosstalk between the peritoneal archimetrotic lesions and the endometrium has been repeatedly described in studies performed in subhuman primates [221, 222]. Sufficient explanations of this phenomenon are still lacking. Our concept that archimetrotic lesions are to be considered as archimetral (“Müllerian”) organoid structures could provide an approach toward an explanation. As outlined before, florid archimetrotic lesions, such as DIA, are subjected to chronic biomechanical stress. In consequence of this ongoing tissue injury and repair process (TIAR), bone-marrow derived stem cells are mobilized and arrested presumably not only in the active lesion but also at other archimetrotic or archimetral sites with the presence of CXCL12 as the ligand to the receptor CXCR4 [160], such as in the uterus, where they are converted into archimetral progenitor cells and enter a proliferative process. A direct cross talk via the circulation between archimetral and archimetrotic tissue involving cellular elements, specific macrophages and chemokines and not necessarily involving the bone marrow loop could also be possible [68, 223].

This ongoing cross talk could be the pathophysiological basis for the notion that women with severe archimetrosis are suffering from a generalized inflammatory status and that archimetrosis could be considered a systemic disease [62, 68, 224–226].

## Conclusions

Based on evolutionary, clinical and experimental evidence, the concept of tissue injury and repair (TIAR) is the most parsimonious explanation for the development of archimetrosis that is initiated at the level of the basal endometrium and induced by uterine hypercontractility in non-conceptual cycles. With the beginning of encephalization during the Cretaceous–Terrestrial revolution and with the parallel evolution of a third myometrial layer, the stratum vasculare, the process of birth had dramatically changed and gave rise to a new mammalian order, the haplorrhine primates, with an inherent predisposition to develop archimetrosis.

The main clinical finding is to be able to define a special risk population due to the high prevalence of primary dysmenorrhea today. Although 60–80% of all women develop premenopausal archimetrosis (adenomyosis/endometriosis), about 10–15% of them are affected at a younger age. Presumably, there is a pathophysiologic continuum in the strength of uterine contractility based on the expression of oxytocin receptors with an evolutionary selection in favor of hypercontractility going back well into the development of menstruating primates.

### Pathogenesis develops in three intertwined processes

1. The traumatization of the archimetra in the area of endometrial–myometrial junction by organ-specific biomechanical functions.
2. The activation of the non-organ-specific, but physiological TIAR process for local production of estradiol and formation of the also non-organ-specific basal morphogenetic complex for attraction of mesenchymal stem cells (MSCs) to the site of trauma.
3. Organ-specific differentiation of MSC into endometrial (ESC) or archimetral stem cells (ASC) and their proliferation and further differentiation into all tissue components of the archimetra such as endometrial epithelium, stroma and metaplastic muscle fibers. Archimetrotic lesions are considered as archimetral (“Müllerian”) organoid structures. In principle, iatrogenic archimetrosis develops in the same way.

Focal and diffuse proliferations of Müllerian tissue destroy the functional morphology of the "junctional zone myometrium" (archimyometrium). In MRI and TVS, these proliferations are seen as "widening" of the junctional zone or "halo" and serve as diagnostic criteria.

There is a high association of uterine archimetrosis with peritoneal archimetrosis. Fragments of basal endometrium containing stem or progenitor cells are disseminated into the peritoneal cavity via the tubes and into the body periphery via the vascular system, forming foci of archimetrosis. They are composed of all archimetral elements ("mini primordial uteri") and persist at sites of chronic mechanical stress.

Early menarche with early onset of regular ovulatory cycles and particularly high contractile activity of the stratum vasculare of the uterus as indicated by primary dysmenorrhea are characteristic for women with a high although, without doubt, individually varying risk for developing archimetrosis. But at the same time, these criteria indicated reproductive health for thousands of years. This is, in our opinion, an important conjecture resulting from our pathogenetic and pathophysiological concept and a translational aspect of our research, in that suppressing hypercontractility as early as possible after onset of menarche and primary dysmenorrhea, a the disease and its sequels could be prevented and health and fertility may be preserved.
